# How Will Nanomedicine Revolutionize Future Dentistry and Periodontal Therapy?

**DOI:** 10.3390/ijms26020592

**Published:** 2025-01-12

**Authors:** Emira D’Amico, Gitana Maria Aceto, Morena Petrini, Chiara Cinquini, Simonetta D’Ercole, Giovanna Iezzi, Tania Vanessa Pierfelice

**Affiliations:** 1 Department of Medical, Oral and Biotechnological Sciences, University “G. d’Annunzio”, via dei Vestini 31, 66013 Chieti, Italy; emira.damico@unich.it (E.D.); gitana.aceto@unich.it (G.M.A.); morena.petrini@unich.it (M.P.); simonetta.dercole@unich.it (S.D.); tania.pierfelice@unich.it (T.V.P.); 2 Department of Surgical, Medical, Molecular Pathologies and of the Critical Area, University of Pisa, Lungarno Antonio Pacinotti, 43, 56126 Pisa, Italy; chiara.cinquini@gmail.com

**Keywords:** periodontitis, nanoparticles, diagnostic activity, antibacterial activity, anti-inflammatory activity, periodontal regeneration

## Abstract

Periodontitis is a prevalent inflammatory disease affecting the supporting structures of the teeth, leading to gum recession, tooth loss, and systemic health complications. Traditional diagnostic methods and treatments, such as clinical evaluation and scaling, often fall short in early detection and targeted therapy, particularly in complex or advanced cases. Recent advancements in nanomedicine offer promising solutions for improving both the diagnosis and treatment of periodontitis. Nanoparticles, such as liposomes, quantum dots, and nanorods, have demonstrated potential in enhancing diagnostic accuracy by enabling more precise detection of periodontal pathogens and biomarkers at the molecular level. Furthermore, nanotechnology-based therapies, including drug delivery systems and antimicrobial agents, offer localized and controlled release of therapeutic agents, enhancing efficacy and reducing side effects compared to conventional treatments. This study reviews the current applications of nanomedicine in the diagnosis and treatment of periodontitis, highlighting its potential to revolutionize periodontal care by improving early detection, reducing treatment times, and enhancing therapeutic outcomes.

## 1. Introduction

Periodontal disease is a chronic inflammation that affects the supporting tissues of the teeth, including the gums, the periodontal ligament, and the alveolar bone. According to the World Health Organization, severe periodontal disease is estimated to affect approximately 19% of the world’s adult population, accounting for over 1 billion cases worldwide [1]. The etiology of periodontal disease is a local inflammatory process driven by subgingival bacteria [2]. Commensal bacteria in the plaque do not cause tissue destruction if controlled by oral hygiene and supported by a healthy diet [3]. Current evidence of the etiology of periodontal disease is the concurrent presence of bacterial dysbiosis in individuals with a dysregulation of the systemic inflammatory response. Dysbiosis is associated with a dysregulated immune response, a decrease in anti-inflammatory bacteria, and an increase in opportunistic or pathogenic species favored by metabolic alterations of the microenvironment. However, the presence of persistent inflammophilic bacterial species also supports the chronicity of damage with the destruction of tooth-supporting tissues and impaired resolution processes [4]. This inflammatory condition affecting the supporting structures of the teeth is often associated with low-grade local or systemic inflammation, which allows the distant transfer of oral bacteria or their components to other distal tissues [5,6,7]. These events support bacterial dysbiosis, the subsequent chronicity of the pathology, and the severity of other systemic chronic conditions [2,4]. It has been shown that there is a dual interaction between periodontal disease and diabetes. Periodontitis has a significant impact on diabetes control, incidence, and complications [8]. The successful treatment of periodontal disease has also led to a significant reduction in glycated hemoglobin [8]. Current therapeutic approaches to periodontitis range from behavioral changes to surgical interventions [9,10]. The first objective is removing subgingival plaque and controlling local and systemic inflammation, followed by tissue regeneration. However, these goals are not easily achieved in most adult patients with aggressive periodontitis [11,12]. Indeed, the American Academy of Periodontology (AAP) and the European Federation of Periodontology (EFP) emphasize the importance of a multidisciplinary approach that considers both oral and systemic pathology, reflecting a more integrated vision of patient care [13]. New therapeutic opportunities may be provided by developing new materials that are functional and adaptable to the individual patient’s pathological condition to avoid the limitations of traditional treatments such as mechanical instrumentation, surgery, and systemic or local antibiotics. The new frontier of these treatments lies in nanomedicine, nanotechnology, and nanosystems, which offer more targeted, efficient, and personalized therapeutic approaches. While nanomedicine focuses on applying nanotechnology to medical treatments, nanotechnology itself encompasses a broader field of science and engineering aimed at manipulating matter at the nanoscale. Nanosystems, on the other hand, refer to integrated, functional structures made from nanoscale components designed to perform specific tasks, often combining the principles of both nanomedicine and nanotechnology for advanced medical applications [14,15,16].

**Figure 1:**
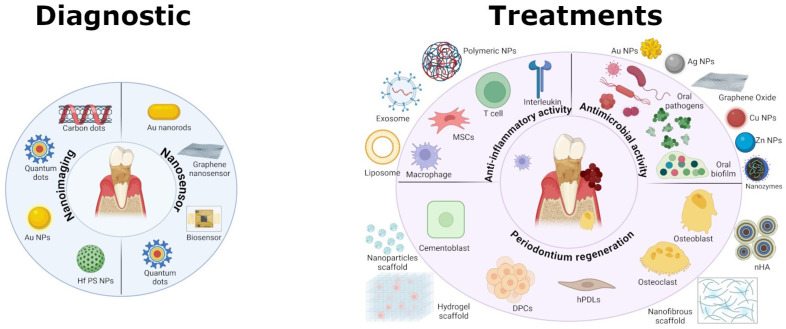
Nanoparticles (NPs) have diverse applications in the diagnosis and treatment of periodontal disease. Diagnostic NPs, such as quantum dots, carbon dots, and gold nanorods, enable highly sensitive and specific detection of periodontal pathogens and biomarkers. Therapeutic NPs, including polymeric NPs, liposomes, metal NPs, graphene oxide, and nanozymes, exhibit antimicrobial, anti-inflammatory, and regenerative properties, supporting tissue healing and preventing disease progression (illustration created using BioRender.com).

The application of nanomedicine and nanotechnologies has been suggested to be a part of the therapeutic arsenal for the treatment of periodontal diseases, mainly periodontitis, with the goal of delivering a sufficient concentration of active molecules at the targeted site and avoiding its distribution in non-specific tissues, consequently decreasing the risk of side effects [17]. However, further research, regulatory approval, and safety evaluations are needed before these technologies can be widely implemented in clinical practice. Although several studies have investigated the characteristics and the effects of nanoparticles (NPs) for treating periodontal diseases, the majority of investigations are performed on in vitro models. The few in vivo experiments have shown that nanomedicine holds great promise for the treatment of periodontal diseases, with positive results in drug delivery, bacterial infection control, and tissue regeneration. While these studies have demonstrated efficacy in animal models, further research is required to fully understand the safety, long-term effects, and clinical applicability of nanomedicine in human periodontal disease treatment. The next steps will likely involve clinical trials to confirm the results seen in animal studies and to determine the most effective nanomedicine formulations for periodontal care [14,18]. This study summarized nanotechnology-adopting strategies for diagnosing and treating periodontitis in terms of antibacterial therapy, anti-inflammatory therapy, and tissue regeneration (Figure 1).

## 2. Nanotechnology for Diagnostic Tools

To effectively treat periodontitis, clinicians must understand not only the clinical signs but also the status, severity, and activity level of disease. Accurate and prompt diagnosis of periodontitis enables clinicians to identify whether the disease is active, stable, or progressing, which directly influences treatment strategy. Examining the full-mouth bleeding score, full-mouth plaque score, recessions, movement, migration, probing depth, clinical attachment level, and bleeding on probing (BoP) are all clinical evaluations that provide a comprehensive understanding of the patient’s periodontal health [19]. However, these methods alone cannot identify the presence of specific pathogens causing the infection, which is also crucial for developing targeted treatments. Nanotechnology also offers advances in early diagnosis and monitoring of periodontal diseases through biosensors and imaging techniques [16]. Nanomaterials can be functionalized with specific molecules (such as antibodies or peptides) that bind to disease markers or pathogens. This allows for highly targeted imaging, which can dramatically improve diagnostic accuracy, especially for detecting specific bacterial infections (Table 1). Peptide-functionalized nanoparticles could improve the specificity of diagnostic imaging agents, making it possible to detect pathogens at very low concentrations in the oral cavity, which would otherwise be missed by traditional imaging techniques. Targeted nanoparticles could be used in fluorescence or optical coherence tomography (OCT) to selectively highlight regions of interest, such as areas of early decay, gum disease, or tumors [15].

### 2.1. Nanotechnology-Enhanced Imaging

The continuous evolution of imaging technologies in dentistry has dramatically improved diagnostic precision, reduced treatment times, and enhanced patient care. From 3D imaging systems like cone-beam computed tomography (CBCT) and intra-oral scanners to innovative artificial intelligence (AI)-driven analysis tools, these advances are reshaping how dental professionals diagnose and treat a variety of oral conditions. As these technologies become more accessible and affordable, they are likely to become integral to routine dental practice, driving further improvements in both patient outcomes and operational efficiency [20]. In imaging technologies, nanomaterials are increasingly employed for enhancing resolution, sensitivity, and the overall effectiveness of diagnostic tools. The innovative use of nanotechnology aims to reduce radiation exposure while producing sharp, detailed pictures, improving dental imaging safety and accuracy. The NPs work at the molecular or nanoscale level, providing unique properties that are difficult to achieve with conventional materials. High atomic number (high-Z)-based nanoparticles such as gadolinium (Gd), ytterbium (Yb), hafnium (Hf), tantalum (Ta), tungsten (W), rhenium (Re), gold (Au), and bismuth (Bi) are being explored as potential contrast agents to significantly enhance the visibility of tissues on X-rays and computed tomography (CT) scans. High-Z elements, due to their high atomic number, absorb X-rays more effectively than other elements commonly found in the body such as carbon, oxygen, nitrogen, and calcium [21]. This property leads to increased X-ray attenuation, providing greater contrast between the high-Z elements and the surrounding tissues [21,22]. Compared to iodinated CT contrast agents, AuNPs have a significantly higher X-ray attenuation. Iodinated agents are excreted rapidly from the body, resulting in a short imaging time [21]. In contrast, AuNPs have a K-edge energy of 80.7 keV, which allows AuNPs to outperform iodinated contrast agents in terms of image quality at the same concentration. AuNPs are less toxic to kidneys than iodinated contrast agents, and thus could potentially offer a safer option for patients, particularly those with renal impairments [22]. Ostadhossein F. et al. described a novel approach combining polymeric silane and hafnium oxide (HfO_2_) nanoparticles (Hf PS NPs) for both diagnostic imaging and therapeutic applications, specifically targeting oral pathogens. Experiments conducted on human tooth samples outside of a living organism demonstrated a significant difference in X-ray absorption between the nanoparticles and the tooth material. A high-affinity, pathogen-selective peptide was used to guide the nanoparticles specifically to the pathogen, allowing for molecularly targeted X-ray imaging. This could provide a more precise method of identifying and localizing the bacterial pathogen in dental applications, especially in the context of caries [23]. Nanomaterials like quantum dots or carbon-based nanomaterials can be designed to fluoresce at specific wavelengths when exposed to light. These materials can be incorporated into fluorescence-based imaging technologies to enable the detection of early-stage dental issues that may not be visible through conventional methods. The unique characteristics of carbon dots (CDs), including their fluorescence properties, low toxicity, and the ability to modify their surface chemistry for selective interactions with bacteria, make them promising candidates for improving the diagnostic and therapeutic management of bacterial infections. Yang J. et al. constructed quaternized CDs that exhibited bacterial-contact-enhanced fluorescence emission. This modification enhanced the selectivity and sensitivity of CDs for detecting Gram-positive bacteria. The quaternized CDs can differentiate between Gram-positive and Gram-negative bacteria using fluorescence signals, facilitating faster diagnosis of bacterial infections [24]. Liu S. et al. focused on CDs doped with nitrogen (N) and chlorine (Cl) elements. These positively charged N, Cl-codoped CDs were found to exhibit high selectivity for Gram-positive bacteria through selective fluorescence imaging, and antibacterial effects. The positively charged surface of the N, Cl-codoped CDs likely facilitates strong electrostatic interactions with the negatively charged membranes of Gram-positive bacteria, contributing to both their selective recognition and bactericidal action [25].

### 2.2. Nano-Biosensors

**Figure 2:**
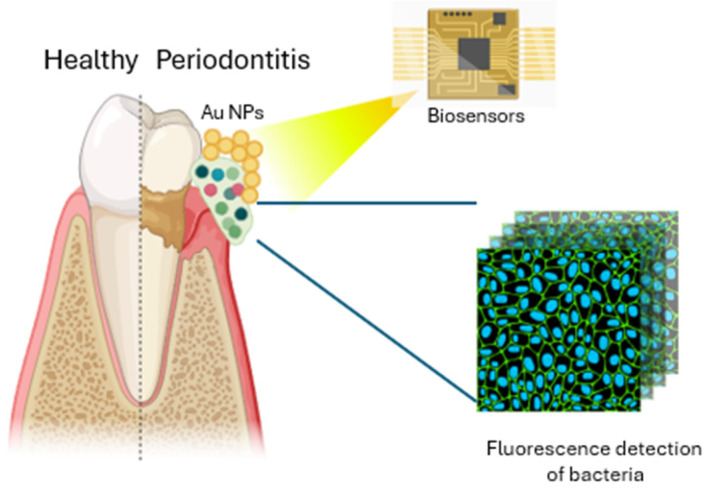
Biosensors incorporating gold nanoparticle (yellow area) can be used to detect harmful bacteria or pathogens responsible for dental diseases like periodontitis. Specific antibodies or aptamers can be used to recognize bacterial antigens (multicolored spheres). When these bacteria are present in the saliva or plaque, they will bind to the functionalized gold nanoparticles (gold spheres, Au NPs), triggering a detectable signal (blue-green fluorescence). (illustration created using BioRender.com).

The combination of nanomaterials and biosensor technologies offers a powerful and flexible approach for the detection and quantification of a wide range of biological and chemical substances, enabling more efficient diagnostic tools for clinical and environmental applications. Metal nanoparticles (MNPs), carbon nanotubes, and quantum dots (QDs) are employed for enhancing the sensitivity and precision of electrochemical biosensors. These materials are integral to the performance of nanosensors, which have gained increasing attention over the last few decades, surpassing other analytical techniques like chromatography and spectrophotometry (Figure 2) [26]. Electrochemical biosensors are especially favored for point-of-care (POC) diagnostics due to their sensitivity, rapid response time, and practicality, making them well-suited for portable, on-site applications to detect a wide range of analytes, including pharmaceuticals, proteins, biomarkers, and pathogens [27,28]. MNPs conjugated with antibodies are a powerful tool in immunomagnetic separation (IMS) for biosensor applications. This technique combines the specificity of antibodies for target antigens with the magnetic properties of metal nanoparticles to efficiently capture and separate biomolecules or cells of interest from complex biological samples [16,28]. Ma D. et al. developed a novel, easy-to-use, low-cost detection platform for monitoring dental health, specifically targeting the detection of tooth lesions caused by dental caries and periodontal diseases. The platform is a wearable mouthguard made of a composite material consisting of gold–silver nanorods (Au@Ag NRs) and poly(dimethylsiloxane) (PDMS), which can visualize the presence of dental lesions through a color change at the affected sites. The color change occurs in response to the presence of hydrogen sulfide (H_2_S) gas, which is produced by bacterial decay at the lesion sites in the mouth. In addition to its sensing capabilities, the Au@Ag NRs–PDMS mouthguard exhibits several desirable characteristics such as mechanical properties for maintaining its integrity during wear, resistance to degradation from chemical exposure, making it suitable for the harsh oral environment, and high biocompatibility [29].

Mannoor M.S. et al. created a highly sensitive, selective, and non-invasive sensing system including graphene nanosensors able to interface with biomaterials such as tooth enamel. The system can detect single-cell bacterial infections and provide wireless remote monitoring, making it a promising tool for healthcare applications. In this system, the graphene network is integrated onto the biomaterial interface, allowing it to conform closely to biological surfaces. This intimate contact ensures that the sensor can pick up even very subtle changes in the environment, such as the presence of bacteria. The graphene nanosensors are transferred to the biological surfaces using a water-soluble silk fibroin platform, which provides a biocompatible and flexible base. Silk fibroin serves as a medium that enables the graphene to be transferred seamlessly, preserving its sensitive properties while ensuring that the system remains biologically safe and able to interact with living tissues. The sensor integrates antimicrobial peptides (AMPs), which enable broadly selective biorecognition. These peptides are tailored to specifically target bacterial cells, enhancing the sensor’s specificity for detecting single-cell bacteria. The combination of graphene, AMPs, and the resonant circuit results in a highly sensitive, selective, and wireless sensor that can be used for a wide variety of applications, from detecting bacteria in oral cavities to monitoring environmental pollutants or tracking health conditions in real time [30].

**Table 1:** Diagnostic nanostrategies for periodontitis.

Nanoparticles	Study Type	Methods	Effects	References
Hf PS NPs	In vitro	Polymeric-silane-conjugated hafnium oxide nanoparticles	Precise method of identifying and localizing the bacterial pathogen using molecularly targeted X-ray imaging	[23]
CDs	In vitro/in vivo (*S. aureus*-infected mouse model)	One-pot solvothermal method to prepare quaternized CDs	Inactivation of Gram^+^ bacteria via disrupting the bacterial walls/membranes	[24]
N,Cl-CDs	In vitro	Synthesized metal-free N,Cl-doped carbon dots using *Impatiens balsamina* L. stems as green precursors in a deep eutectic solvent (DES)	High selectivity for Gram^+^ bacteria through selective fluorescence imaging, and antibacterial effects	[25]
Au@Ag Nanorods–PDMS	In vitro/in vivo (human)	Nanomaterial consisting of gold–silver nanorods (Au@Ag NRs) and PDMS	Visualization of the presence of dental lesions through a color change at the affected sites	[29]
Functionalized graphene with AMPs	In vitro	Graphene nanosensors functionalized with dodecapeptide graphene, triglycine linker, and the AMP odorranin-HP (OHP)	Highly sensitive, selective, and wireless sensor that can be used for detecting bacteria in oral or tracking health conditions in real time	[30]

## 3. Nanomaterials for Antibacterial Therapy

**Figure 3:**
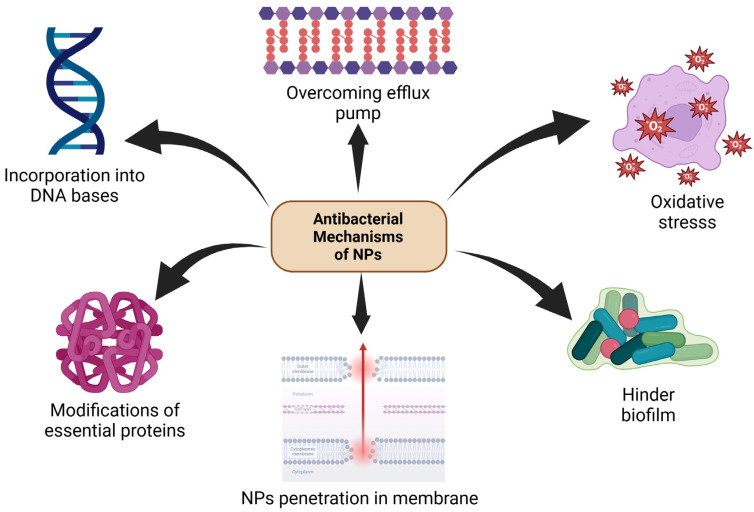
Antibacterial mechanisms of nanoparticles (NPs) include incorporation into DNA bases, overcoming efflux pumps, inducing oxidative stress, hindering biofilm formation, modifying essential proteins, and penetrating bacterial membranes (illustration created using BioRender.com).

The first aim of treating periodontal disease is to eliminate subgingival debris and plaque to reduce the bacterial load. Although the administration of antibiotics represents a therapeutic strategy for treating the acute manifestation of periodontitis, they fail to support the long-term health of periodontal tissues, and they do not provide tissue regeneration. Moreover, the emergence of antibiotic resistance in bacteria is a significant global health challenge [31]. Nanomaterials are emerging as a promising approach to address antibiotic resistance due to their ability to evade existing resistance mechanisms adopted by bacteria [32]. Resistance in bacteria can be intrinsic, which refers to the inherent ability of bacteria to resist the effects of antibiotics due to their natural characteristics or structural features [33,34]. Gram-negative bacteria possess an outer membrane that limits the entry of certain antibiotics, making them inherently resistant to many drugs. Many bacteria have evolved efflux pumps that can actively transport a range of antimicrobial agents out of the cell, decreasing drug accumulation and effectiveness [33,34]. *Pseudomonas aeruginosa* is intrinsically antibiotic-resistant due to its impermeable outer membrane and efficient efflux pumps [35]. On the other hand, extrinsic resistance refers to the acquired ability of bacteria to resist antibiotics. The development of resistance is a complex process influenced by genetic, environmental, and selective factors [33]. Spontaneous mutations in target genes can lead to changes that prevent antibiotic binding, such as alterations in ribosomal RNA or penicillin-binding proteins. Bacteria can acquire resistance genes from other bacteria through mechanisms like transformation, transduction, or conjugation, leading to the spread of resistance traits. *Staphylococcus aureus* can acquire methicillin resistance (MRSA) through horizontal gene transfer of the mecA gene [36]. Enterobacteriaceae can develop resistance to carbapenems by acquiring carbapenemase genes [37]. Some bacteria can produce enzymes that can inactivate antibiotics (e.g., beta-lactamases that break down penicillin) [38]. Differences in structure lead to variations in resistance mechanisms between Gram-negative and Gram-positive bacteria. Gram-negative bacteria usually employ all mechanisms: reducing drug uptake, altering drug targets, inactivating drugs, and actively expelling drugs. Gram-positive bacteria less frequently employ reduced drug uptake due to the absence of an LPS outer membrane and lack specific efflux capabilities [33]. In bacterial infections, biofilms play a critical role, providing a protective environment that shields bacteria from the action of antibiotics. The cells within biofilms are often in a dormant state, making them less susceptible to treatments [39]. Nanoparticles are being explored as alternatives to traditional antibiotics due to their unique properties and mechanisms of action. Nanomaterials can be highly effective in controlling infection due to their small size, enhanced surface area, shape, and ability to deliver therapeutic agents directly to the infection site (Table 2) [32]. Various types of nanoparticles employ multiple mechanisms simultaneously to fight microbes, such as nitric-oxide-releasing nanoparticles (NO-NPs), chitosan-containing nanoparticles (chitosan-NPs), and metal-containing nanoparticles (metal-NPs) (Figure 3) [40,41]. This multifaceted approach to antimicrobial action hinders the microbes from developing resistance.

### 3.1. Nanoparticle–Membrane Interaction

The interaction between the nanoparticle surface and the bacterial membrane is the starting point for the antimicrobial action of NPs. Their small size allows nanoparticles to penetrate biological barriers effectively. Metal-NPs easily bypass Gram-negative bacteria’s lipopolysaccharide (LPS), through their channel proteins [42]. Yang Y. et al. demonstrated a size-dependent effect of gold nanoparticles (AuNPs) on bacterial LPS in promoting neutrophil uptake. Smaller (10 nm) AuNPs promoted the response of neutrophils more than larger (40 and 100 nm) AuNPs [43]. It is well known that smaller nanoparticles have a larger surface-area-to-volume ratio. This means that more of the nanoparticle’s surface is exposed to the surrounding environment [44]. Results from various studies have showed how the size of GO sheets impacts the effectiveness of their antimicrobial properties in different contexts [45,46]. Larger GO sheets have a greater capacity to wrap around bacterial cells, isolating them from their environment and inhibiting their growth in suspension [45,46] On the other hand, their interaction with bacteria differs when GO sheets are immobilized on a surface. In this case, smaller GO sheets may exhibit greater antimicrobial activity due to two main factors. Smaller sheets have a higher surface-to-volume ratio, leading to more potential interaction sites with bacterial cells. Smaller sheets have higher defect density, which can increase their reactivity and potentially enhance their antimicrobial properties through oxidative mechanisms [46,47]. Another relevant toxicity factor responsible for size-dependent antibacterial activity is that smaller nanoparticles dissolve more quickly than larger ones. The rapid dissolution leads to a more rapid release of metal ions [44]. Skandalis N. et al. observed through scanning electron microscopy (SEM) that smaller (40 nm) silver nanoparticles (AgNPs) induced stronger membrane damage in *E. coli* after 10 h than larger ones (58 nm) [48]. Recently, Zhang Y. et al. proposed the ultra-small gold nanoclusters (AuNCs) composed of 25 gold atoms and 18 thiolate ligands. High-resolution transmission electron microscopy (TEM) showed that AuNCs displayed a homogeneous and well-dispersed distribution, and the particle size of AuNCs ranged from 1.5 to 4.0 nm with an average diameter of 2.49  ±  0.30 nm. The results indicated that AuNCs induced lysis of the *Fusobaterium nucleatum* membrane potential with consequential cell wall integrity damage [49]. Different studies demonstrated that the shape of nanoparticles is another critical parameter with respect to antibacterial activity [50,51,52]. In their study, Acharya et al., through FE-SEM images, showed structural damage to bacterial cell walls upon treatment with spherical silver nanoparticles (AgNP-sp) but not with rod-shaped silver nanoparticles (AgNR) [50]. In another study, the same authors observed the highest bacterial death when Gram-positive and Gram-negative bacteria were treated with nanospheres, compared to nanorods, nanotriangles, and nanohexagons [51]. Hong X. et al. fabricated AgNPs having three different shapes, via a microwave-assisted method, and tested them against various bacteria species. The authors observed the weakest antibacterial activity in silver nanowires, compared to silver nanocubes and silver nanospheres, due to the lower amount of contact between silver nanowires and the bacterial membrane [52]. Electrical potential, or “zeta potential”, is a key parameter in determining the stability and behavior of particles in a liquid medium and affects their antibacterial activity. Strong zeta potentials promote a strong interaction, causing membrane disruption, bacteria flocculation, and a reduction of viability [53,54]. Zhang Y. et al. synthesized ultra-small gold nanoclusters (AuNCs) by a simple one-pot method, with a zeta potential of −38.8 mV. The results showed that the growth of *Fusobacterium nucleatum* was significantly hampered, and cell wall integrity was strongly damaged via a membrane depolarization mechanism. Thus, the zeta potential, which depends on the surface charge, is fundamental for the stability of nanoparticles in suspension and affects the initial adsorption of nanoparticles onto the cell membrane. Charge is crucial in bacterial resistance due to its influence on various cellular processes and interactions with antimicrobial agents. Cationic nanoparticles have been demonstrated to effectively depolarize and potentiate the bacterial membrane, facilitating the direct translocation of NPs to the cytosol region [55]. Inside cells, cationic NPs interact by high affinity with DNA which is negatively charged, inducing conformational changes and disrupting bacterial replication [56]. In their study, Haidari H. et al. tested newly synthesized, highly monodispersed, small (<3 nm) polycationic silver nanoclusters (pAgNCs) against a range of common Gram-negative and Gram-positive oral pathogens and against oral biofilm. The pAgNCs displayed greater antibacterial efficacy than similar-sized negatively charged silver nanoparticles or than ciprofloxacin [57]. The synthesis of these pAgNCs allowed them to overcome the limits of anaerobic environment. Indeed, the dissolution of Ag^+^ ions is an oxidation process, and the release rate is highly dependent on the presence of molecular oxygen. The pAgNCs also showed a strong capacity to significantly delay the development of bacterial resistance in anaerobic bacteria commonly found in dental infections, such as *Fusobacterium nucleatum* and *Streptococcus sanguinis* [57]. Caudill E. et al. have observed an enhanced electrostatic attraction between positively charged gold nanoparticles functionalized with cationic branched polyethylenimine (bPEI-AuNPs) and Gram-positive bacteria due to the presence of negatively charged groups on the cell surface, such as teichoic acids [58].

### 3.2. Nanoparticles Target Efflux System

Nanoparticles offer different approaches to overcoming efflux pumps as a defense mechanism adopted by bacteria: (1) creating a competition between substrate and antimicrobial agents; (2) downregulating the expression of efflux pumps; (3) blocking the efflux pumps by a designed molecular plug; (4) interacting directly with efflux pumps, by blocking their active sites or altering their conformation; and (5) indirectly modulating the expression or activity of efflux pumps [59]. Sobhanipoor M.H. et al. observed a reduction in the efflux activity in enterococcal strains treated with zinc oxide nanoparticles (ZnONPs) [60]. In the study conducted by Christena L.R. et al., copper nanoparticles (CuNPs) exhibited a significant efflux-inhibitory effect in wild-type strains of both *Staphylococcus aureus* and *Pseudomonas aeruginosa* and in drug-resistant mutant strains of *Staphylococcus aureus.* The authors proved that the antibacterial effect is due to Cu(II) ions released from the CuNPs more than the nanoparticle itself [61]. Several metal oxide nanoparticles have been suggested for their combination with thiolated chitosan to tackle the multi-drug resistance problem in bacteria by blocking the efflux pump [62]. Iqbal G. et al. exploited the physical–chemical characteristics of some metals to prepare thiolated-chitosan-coated-cobalt-doped zinc oxide nanoparticles (Co–ZnO), which were then able to induce inhibition of the efflux pump in drug-resistant mutant strains of *Staphylococcus aureus* [63]. Efflux pumps, often the targets of the nanoparticles employed for combating biofilm-related infections, are characterized by a selective and orchestrated drug outgo [59]. In a study, it was observed that ZnONPs inhibit biofilm formation and virulence factor production in *Pseudomonas aeruginosa,* by inducing the zinc cation efflux pump (Czc operon) at a genetic level and regulating key transcriptional factors (porin gene opdT and type III repressor ptrA), which directly blocks the efflux pump [64].

### 3.3. Nanoparticle-Induced Oxidative Stress

Nanomaterials can function through various mechanisms that differ from traditional those of antibiotics, such as generation of ROS. Oxidative stress has been suggested as the main mechanism in the antimicrobial activity of bacterial cells exposed to GONPs. The high defect densities on the carbon structure act as active sites for oxygen molecules to adsorb onto the GO nanosheet surface. The adsorbed oxygen molecules become more reactive due to their interaction with the GO surface [46,65]. These reactive oxygen molecules can then react with other molecules, including those in the cell membrane of bacteria, to generate highly reactive species like hydroxyl radicals. Perreault F. et al. observed a flattened and deformed bacterial shape, indicative of compromised cell integrity, in *E. coli* deposited on GO-coated surfaces. The same authors also observed that GO nanosheets can oxidize lipid molecules and glutathione (GSH) enzymes, demonstrating their intrinsic oxidative potential. This oxidative effect on glutathione was found to be size-dependent. Smaller GO sheets (0.01 μm^2^) induce greater oxidation (71%) compared to larger ones (0.65 μm^2^, 49%) [46]. Panda S. et al. described the molecular mechanism behind the antibacterial effect of GO nanosheet metal systems on Gram-negative bacteria *E. coli*. GO possesses abundant oxygen-containing functional groups like hydroxyl, epoxy, and carboxyl on its surface, which make GO an excellent electron acceptor. When GO comes into contact with a bacterial cell, it can draw electrons from the cell membrane. The electron transfer to GO triggers the production of ROS within the bacteria [66]. Interestingly, it was found that cobalt as a dopant was able to increase the photodynamic and photothermal activity of Co–ZnO. Upon excitation in light, these nanoparticles were able to generate ROS with an increased quantum yield, and to generate heat, because of the magnetic nature, thus helping to kill more drug-resistant mutant strains of *Staphylococcus aureus* [63]. Gurunathan S. et al. noted that the levels of ROS in GO and reduced graphene oxide (rGO)-treated *Pseudomonas aeruginosa* were 3.8-fold and 2.7-fold higher, respectively, compared to the level of ROS in control cells [67]. After 24 h from treatment, the same authors observed DNA fragmentation in cells treated with GO, but not in bacteria treated with rGO, which suggests that cells require longer exposure to rGO to induce DNA fragmentation or that the mechanism of cell death caused by rGO could be different from that of GO after ROS production [67]. In particular, the generation of oxidative stress is the main mechanism of metal NPS to the detriment of essential cellular components, such as proteins and nucleic acids. In a recent study, Wang Y. et al. synthesized stable gold nanoparticles (AuNCs) that are protected with 6-mercaptohexanoic acid (MHA). These nanoparticles consisted of 25 gold atoms and 18 thiolate ligands, formed through a one-pot reduction process converting gold (III) to gold (0). The results showed the antibacterial properties of these Au_25_ NCs against both Gram-negative and Gram-positive bacteria after the disruption of antioxidant defense systems by the increase of intracellular ROS level and decrease of glutathione (GSH) [68]. The same authors also observed that the increase in ROS production was greater in Gram-negative bacteria than in Gram-positive ones [68]. Similarly, Zhang Y. et al. observed an increase of the level of ROS in *Fusobacterium nucleatum* after treatment with ultra-small gold nanoclusters (AuNCs) consisting of 25 gold atoms and 18 thiolate ligands formed through a one-pot reduction process [49]. The generation of ROS is the mechanism underlying the antibacterial action of nanozymes (NZs), which refer to nanomaterials that have catalytic properties like natural enzymes. For this reason, recently, NZs have significantly advanced research in the fields of periodontics, specifically for the maintenance of periodontal health. In particular, NZs are employed for disrupting dental plaque, a complex biofilm composed of diverse bacterial species, which is notoriously recalcitrant to traditional antimicrobial agents. The exopolysaccharide (EPS) matrix acts as a protective shield encasing the microbial community, hindering penetration by agents and thus limiting their efficacy. Furthermore, the acidic microenvironment created within the biofilm promotes enamel demineralization, leading to dental caries. Nanohybrid systems are developed to exploit the acidic environment within oral biofilm to be activated to allow NZs to convert hydrogen peroxide (H_2_O_2_), produced by bacteria, into free radicals, remaining within the three-dimensional structure of dental plaque. The combination of nanozymes and H_2_O_2_ synergistically degrades EPS and eliminates bacteria-forming biofilm [69]. Huang Y. et al. exploited the pathological (sugar-rich/acidic) conditions using a nanohybrid system to increase intrinsic H_2_O_2_ production and trigger pH-dependent ROS generation for efficient biofilm virulence targeting. The nanohybrid contains glucose–oxidase (GOx) that catalyzes glucose present in biofilms to increase intrinsic H_2_O_2_, which is converted by iron oxide nanoparticles with peroxidase-like activity into ROS in acidic pH. The authors developed dextran-coated iron oxide nanozymes (Dex-IONP) that display strong catalytic peroxidase-like activity at acidic pH values, able to target biofilms with high specificity and to prevent severe damage without impacting surrounding oral tissues in vivo. This system selectively kills the pathogenic bacteria while sparing commensal bacteria. Furthermore, compared to chlorhexidine (positive-control), which disrupted oral microbiota diversity, the nanohybrid had significantly higher efficacy without affecting soft tissues and the oral–gastrointestinal microbiomes, while modulating dental-health-associated microbial activity in vivo [70]. Gao L. et al. also developed catalytic nanoparticles (CAT-NPs) with peroxidase-like activity to target and disrupt plaque biofilm. CAT-NPs containing biocompatible Fe_3_O_4_ were designed to generate free radicals by converting H_2_O_2_. Additionally, the generation of these powerful free radicals is specifically triggered by acidic conditions, which are prevalent within dental plaque [70]. This targeted approach ensures that the action of CAT-NPs/H_2_O_2_ remains limited, minimizing potential harm to healthy oral tissues. Wang Y. et al. designed iron-based nanozymes (IONzymes) and iron sulfide nanozymes (ISNzymes) with peroxidase-like activity catalyzing the generation of free radicals from H_2_O_2_, which is produced by *S. gordonii*, and the consequent radicals disrupting the biofilm matrix [71].

### 3.4. Combination of Therapies

The properties of nanomaterials allow us to overcome some of the challenges of the strategies employed for combating periodontal diseases. Photodynamic therapy (PDT), an emerging approach that involves photosensitizers, light, and molecular oxygen, has shown promise for fighting periodontitis [72]. However, PDT does not always lead to the desired therapeutic outcomes, since some photosensitizers have strong hydrophobic cores, making them difficult to absorb efficiently by periodontal pathogenic bacteria [72]. To overcome this limitation, Li Z. et al. have developed a strategy to enhance the solubility and bacterial adsorption of a hydrophobic photosensitizer chlorin e6 (Ce6). They achieved this by conjugating Ce6 with a cationic cell-penetrating peptide known as TAT. To further optimize the treatment, the TAT–Ce6 conjugate was used to create self-assembled nanoparticles that efficiently load tinidazole (TDZ), a conventional antibiotic. The synergistic combination of PDT and antibiotic therapy, delivered through advanced nanoparticle technology, led to a great inhibitory effect against periodontal pathogens in vitro and in vivo [73]. In another study, Sun X. et al. combined the photosensitizer chlorin e6 (Ce6), the fluorescent dye coumarin 6 (C6), and magnetic iron oxide nanoparticles (Fe_3_O_4_). The co-loading of Ce6 and C6 enabled real-time antibacterial PDT monitoring by ratio emissions with the same wavelength. In contrast, Fe_3_O_4_ with a magnetic field enabled the targeting of infection sites by eliminating multispecies oral biofilm [74]. Cuprous oxide (Cu_2_O), a promising material for photodynamic therapy (PDT), suffers from a major drawback in the rapid recombination of electrons and holes. This limits its effectiveness in generating ROS. To address this issue, He Y. et al. have developed a novel nanosystem (Cu_2_O@rGO) via the in situ growth of Cu_2_O on reduced graphene oxide (rGO) sheets [75]. rGO acts as an electron trap able to capture photoexcited electrons from Cu_2_O, preventing their recombination with holes. rGO facilitates the rapid transfer of electrons away from Cu_2_O. The incorporation of rGO significantly boosts the photocurrent of Cu_2_O@rGO, leading to a higher generation of charge carriers and improved electron–hole separation, demonstrating enhanced antibacterial rates against both *E. coli* and *S. aureus* [75]. Periodontal disease often requires surgical intervention, and guided tissue regeneration (GTR) is a technique that uses membranes to guide tissue growth and healing. However, these membranes can be susceptible to bacterial infection, which can hinder the healing process and lead to complications. To address this issue, Seo N. et al. developed a new type of membrane using polycaprolactone (PCL), a biodegradable polymer, and zinc oxide (ZnO) nanoparticles. The PCL/ZnO membranes showed significantly reduced bacterial adhesion of a common oral bacteria such as *Porphyromonas gingivalis*, and, importantly, the ZnO nanoparticles did not negatively impact the growth of osteoblasts. This study suggests that PCL/ZnO membranes have the potential to improve the success of GTR procedures by preventing bacterial infection and promoting tissue regeneration [76]. Nanofiber technology holds immense potential for developing innovative periodontal therapies. Researchers are exploring various approaches, including DCH-loaded nanofibers for inhibiting pathogens and promoting healing, PCL-loaded ZnO nanofibers for enhanced bone regeneration [77,78], and SPEEK-loaded nanofibers incorporating functionalized zirconia nanoparticles and curcumin for sustained drug release, improved cell viability, and wound healing [79]. In another study, it was observed that the combination of two subsequent layers of nanoparticles characterized by osteoconductive (nHA) and antibacterial bimetallic nanocomposite (nZnO:Ag) inhibited bacterial growth without causing major toxic effects towards osteoblastic cells, and therefore may constitute a promising solution for GTR procedure [80]. Lin J. et al. designed a novel hybrid hydrogel system that combines antibiotic therapy with photothermal treatment. The researchers developed a near-infrared light (NIR)-activated hybrid hydrogel that allows the release of antibacterial drugs and activation of photothermal treatment. Such antibiotics rapidly eliminate periodontal pathogens in the periodontal pocket, and the photothermal treatment maintains low bacterial retention after the drug therapy [80]. Zhao C. et al. explored the use of carbon dots (CDs), specifically perilla-derived carbon nanodots (CNDs), as photosensitizers for antibacterial therapy, combined with near-infrared (NIR). These CNDs exhibited NIR absorption and emission, which is a critical feature for their role in PDT. NIR light is advantageous because it penetrates tissues more effectively than visible light, which could be useful in dental practice. Antibacterial activity measurement showed that the CNDs could inactivate 99.99% of *S. aureus*, *E. faecalis*, and methicillin-resistant *S. aureus* under 660 nm light irradiation for 5 min, while for the Gram-negative bacteria, the bactericidal efficiency was lower than 50%. Intracellular analysis showed that the antibacterial mechanism was due to the ROS generated on the surface of bacteria membranes upon NIR excitation, as well as the hydrophobic interaction between the hydrophobic groups and Gram-positive bacteria membranes [81].

### 3.5. Targeted Drug Delivery

**Figure 4:**
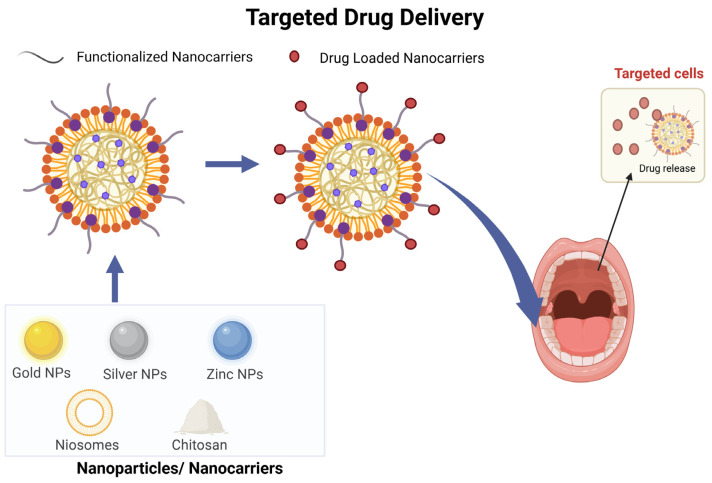
Targeted drug delivery using functionalized nanocarriers loaded with therapeutic agents. Nanocarriers, such as gold nanoparticles (NPs), silver NPs, zinc NPs, niosomes, and chitosan, are designed to deliver drugs specifically to targeted cells, enhancing treatment efficacy (illustration created using BioRender.com).

The use of nanotechnology-based carriers allowed the delivery of the drugs directly to the infection site, leading to a double advantage (Figure 4). Different studies focused on the synergistic activity of ZnONPs with more than 25 different antibiotics against *S. aureus* and *E. coli* have concluded that ZnONPs can enhance the antibacterial activities of penicillin, cephalosporins, aminoglycosides, glycopeptides, macrolides, lacosamide, gentamicin, clarithromycin, ofloxacin, and ceftriaxone and tetracycline [82,83,84,85]. Gold nanoparticles have a stable surface for binding various antibiotic agents and may significantly increase the antibacterial effect of drugs by enhancing contact with bacterial cell walls [86]. Antibacterial activity of vancomycin-capped gold nanoparticles against vancomycin-resistant *Enterococcus* and *E. coli* was 64 times greater than that of vancomycin alone [87]. Nanoparticles can be designed to deliver antibiotics directly to infected cells, reducing the required dosage and minimizing side effects. Saeidi Z. et al. proposed a local dosage form, a thermosensitive gel containing clindamycin niosomes and solid lipid nanoparticles loaded with fluconazole (FLZ), for treating oral infections due to *Candida albicans* and Gram-positive bacteria. The local absorption of clindamycin and fluconazole directly in the oral cavity reduces the amount of them needed, and reduces the systemic side effects such as diarrhea, vomiting, stomach upset, and rush [88]. The results of a recent study demonstrated that the anti-biofilm activity of CuNPs and ZnONPs combined with gentamicin in their lowest concentrations was more efficient than the antibiotic itself. In this study, SEM images showed that CuNPs and ZnONPs used in combination with gentamicin had the highest antibacterial activity when compared with treatment with CuNPs, ZnONPs, and antibiotics alone [89]. The compound with a 50% reduction in ampicillin dosage has a bactericidal activity two times stronger than the antibiotic alone, according to Chamundeeswari M. et al., who created the chitosan-capped gold nanoparticles with ampicillin. MIC values were determined to be 27.4 μg/mL for *E. coli* and 20.6 μg/mL for *S. aureus* and *K. mobilis* when compared to free ampicillin [90]. Ampicillin was employed by Chavan C. et al. as a reducing and capping agent to create gold nanoparticles that were ampicillin-coated. Amp-AuNPs build up on the bacterial surface and lead to the formation of membrane-level holes that allow them to enter the cell. Amp-AuNPs have demonstrated efficacy against ampicillin-resistant *E. coli*, and due to their strong adhesive qualities, they can prevent the development of biofilm [91]. Payne J.N. et al. demonstrated that the conjugation of kanamycin with AuNPs (kan-AuNPs) led to dose-dependent activity with a broad spectrum and minimum inhibitory concentration, lower than antibiotic itself. In this study, the resulting CC50 strongly indicated that Kan-AuNPs would be efficacious in vivo [92]. Existing data suggest that nanoparticles can be used to locally deliver drugs and to protect them from pH and enzymatic degradation in the periodontal lesion [93]. Wang L. et al. designed a novel self-assembled, dual-responsive, and dual-drug-loading nanocarrier system, which included a hydrophobic lipid core formed by 1, 2-Distearoyl-sn-glycero-3-phosphoethanolamine-poly(ethylene glycol) (DSPE-PEG) loaded with alpha-lipoic acid (ALA), and a hydrophilic shell comprising a poly(amidoamine) dendrimer (PAMAM) that electrostatically adsorbed minocycline hydrochloride (Mino). This unique design allows the controlled release of antioxidant/ALA under lipase stimulation from periodontal pathogens and antimicrobial/Mino under the low pH of the inflammatory microenvironment [93]. Another critical challenge for periodontitis therapy is thoroughly eliminating the dental-plaque biofilm, particularly penetrating the deep periodontal tissue without disturbing the commensal microflora of the oral cavity. Tong F. et al. constructed an Fe_3_O_4_ magnetic nanoparticle-loading minocycline (FPM NPs) using a co-precipitation method. The multifunctional nanoparticles allowed for improved drug penetration and exhibited intense anti-biofilm activity by disrupting the integrity of the bacterial biofilm. The periodontal inflammation recovered well after FPM NP treatment in rat models, demonstrating good biocompatibility [94]. Comorbidity often occurs in patients having periodontitis, thus representing a double challenge [95]. Xu S. et al. proposed a novel approach for treating the complex relationship between periodontitis and hypertension, by combining multiple therapeutic strategies in a single delivery system. In this study, a controlled-release composite hydrogel approach was developed with dual antibacterial and anti-inflammatory activities. Specifically, a dual antibacterial hydrogel (CS-PA) has been fabricated by cross-linking chitosan (CS), which displays inherent antibacterial features, with a peptide (AMP)-modified polyethylene glycol (PEG). For long-term anti-inflammatory effects, curcumin has been incorporated into nanoparticles (CNP) and then placed in the hydrogel. CS-PA/CNP administered to the gingival sulcus in a mouse model of periodontitis complicated with hypertension showed a beneficial therapeutic impact on both periodontitis and hypertension at the same time [96].

**Table 2:** Antimicrobial nanotherapeutic strategies for periodontitis treatment.

Nanoparticles	Study Type	Methods	Effects	References
AuNPs	In vitro	LPS selectively synergize with 10 nm AuNPs	AuNPs and LPS augment cellular responses in neutrophils and induce a canonical process of NET formation	[43]
AgNPs	In vitro	Biological pathway using various plant extracts to produce AgNPs	Antibacterial efficacy against *E. coli* and *P. aeruginosa*, *B. subtilis* and *S. epidermidis* by membrane damage	[48]
AuNCs	In vitro/in vivo (male C57 BL/6 mice)	AuNCs composed of 25 Au atoms and 18 thiolate ligands with ultra-small structure	Lysis of the *F. nucleatum* membrane, ROS generation, and inhibition of biofilm formation	[49]
AgNP-sp, AgNR	In vitro	Spherical silver nanoparticles (AgNP-sp)	Structural damage to bacterial cell walls of *K. pneumoniae*	[50]
AgNPs	In vitro	AgNPs in the form of nanocubes, nanospheres, and nanowires prepared via microwave-assisted method	Nanocubes and nanospheres showed stronger antibacterial activity than the nanowires with low specific surface areas against *E. coli*	[52]
AuNPs	In vitro	AuNPs characterized by face-centered cubic lattice structures and truncated-octahedron morphology	Reduction of *F. nucleatum* growth and damage of cell wall integrity by membrane depolarization mechanism	[55]
pAgNCs	In vitro	Highly monodispersed, ultra-small (<3 nm) pAgNCs	Penetration of pAgNCs in the bacterial cell membrane of *F. nucleatum* and *S. sanguinis*	[57]
bPEI-AuNPs	In vitro	Interaction of cationic bPEI-AuNPs with wall teichoic acids of Gram-positive bacterial cell walls	Interaction and damage of bacterial wall of *B. subtilis*	[58]
ZnONPs	In vitro	Green synthesis from natural sweetener *S. rebaudiana*	Action on efflux activity of Enterococci	[58]
CuNPs	In vitro	110 nm casein-stabilized CuNPs	Action as efflux pump inhibitor and anti-biofilm agent on *S. aureus*	[61]
Co–ZnO	In vitro	Synthesis of 40–60 nm Co–ZnO particles by modified co-precipitation method	Photo-inactivation and efflux pump inhibition of methicillin-resistant *S. aureus*	[63]
GONPs	In vitro	Natural shellac-derived GO coatings	Draw-up of electrons from the cell membrane and transfer to GO with consequent ROS production	[66]
GONPs, rGONPs	In vitro	GO dispersion obtained by sonication of graphite (Gt) powders (20 µm)	Loss of cell viability, induced oxidative stress, and DNA fragmentation on *P. aeruginosa*	[67]
Au_25_ NCs	In vitro	Ultra-small gold nanoclusters Au_25_	Destruction of membrane integrity, disruption of antioxidant defense system, metabolic inactivation, DNA damage on *E. coli*	[68]
Dex-IONP, Dex-IONP-GOx	In vitro	Dextran-coated iron oxide nanozymes (Dex-IONP) that display strong catalytic peroxidase-like activity at acidic pH values	Nanohybrid system to increase intrinsic H_2_O_2_ production and trigger pH-dependent ROS generation to kill pathogenic bacteria	[70]
IONzymes, ISNzymes	In vitro	Dextran-coated iron oxide nanozymes (Dex-IONP) that display strong catalytic peroxidase-like activity at acidic pH values	Combination of iron-based nanozymes and H_2_O_2_ provide elimination of oral biofilm	[71]
Ce6, TAT–Ce6 NPs	In vitro/in vivo (female Sprague–Dawley rats)	TAT–Ce6 self-assembled nanoparticles for loading TDZ	Synergistic anti-periodontitis effects of PDT and antibiotic therapy: killing of *P. gingivalis* and the reduced adsorption of alveolar bone in rat	[73]
Ce6, C6, and Fe_3_O_4_ NPs	In vitro	Ce6 and C6 co-loaded into the Fe_3_O_4_–silane core–shell structure to form multifunctional nanoparticles	Strong anti-biofilm activity against *S. sanguinis, P. gingivalis*, and *F. nucleatum,* with magnetically targeting capacities	[74]
Cu_2_O@rGO	In vitro	Nanosystem designed via the in situ growth of Cu_2_O on rGO	Generation of charge carriers and improved electron–hole separation showed enhanced antibacterial rates against *E. coli* and *S. aureus*	[75]
PCL/ZnO	In vitro	Membrane using polycaprolactone (PCL), a biodegradable polymer, and zinc oxide (ZnO) nanoparticles	Inhibition of bacterial adhesion of *P*. *gingivalis* without affecting the viability of osteoblasts	[76]
Au NBPs	In vitro	Mixing of mesoporous silica-coated Au NBPs (Au NBPs@SiO_2_) with gelatin methacrylate (GelMA-Au NBPs@SiO_2_) to deliver minocycline	Higher antibacterial efficacy of the antibiotic and photothermal treatment against *P. gingivalis*	[97]
SPEEK + NH_2_–ZrO_2_ + Cur	In vitro	Amine-functionalized zirconia-nanoparticle-loaded curcumin-incorporated SPEEK nanofibrous scaffolds	Antibacterial activity against *S. oralis*	[79]
CNDs	In vitro	Combination of CNDs with near-infrared	Inactivation of *S. aureus*, *E. faecalis*, and methicillin-resistant *S. aureus* due to ROS generation	[81]
SLNs	In vitro	Thermosensitive gel formulations containing clindamycin-loaded niosomes and solid lipid nanoparticles (SLNs) loaded with fluconazole (FLZ)	The gel formulation presented a slower release of both drugs compared to niosomes and SLN suspensions	[88]
CuNPs and ZnONPs	In vitro	CuNPs and ZnONPs combined with gentamicin	Stronger anti-biofilm activity of CuNPs and ZnONPs combined with gentamicin in their lowest concentrations than antibiotic itself	[89]
C-AuNp-Amp	In vitro	Chitosan-capped gold nanoparticles coupled with ampicillin	Better activity of C-AuNp-Amp compared to free ampicillin	[90]
Amp-AuNPs	In vitro	Synthesized ampicillin-capped gold nanoparticles (Amp-Au NPs)	Amp-AuNPs show successful accumulation onto the surface of the bacterial cell, as a result of which pores were formed into the bacterial membrane of *E. coli*	[91]
Kan-AuNPs	In vitro	Conjugation of kanamycin on the surface of AuNPs	Significant reduction in the MIC of Kan-AuNPs compared to free kanamycin against *S. bovis*, *S. epidermidis*, *E. aerogenes*, *P. aeruginosa*	[92]
DPP, DPPLM NPs	In vitro/in vivo (diabetic rat)	Self-assembled, dual-responsive, and dual-drug-loading nanocarrier system with minocycline loading	Co-delivery of antimicrobial/Mino and the antioxidant/ALA, disruption of dental-plaque biofilms, and suppression of periodontal bone loss	[93]
FPM NPs	In vitro/in vivo (male Wistar rats)	Fe_3_O_4_@PDA nanocomposites with minocycline loading	Robust antibacterial effect against *S. sanguinis*, *F. nucleatum*, and *P. gingivalis*, high biocompatibility, and low systemic toxicity of FPM NPs	[94]
CS-PA/CNP	In vitro/in vivo (mouse model of periodontitis complicated with hypertension)	CS with antibacterial properties cross-linked with AMP-modified PEG to form a dual antibacterial hydrogel (CS-PA) with curcumin loaded into CNP	Co-treatment of periodontitis and hypertension, and drug delivery platform to provide combinatorial therapeutic options	[96]

## 4. Nanotechnology for Anti-Inflammatory Therapy

**Figure 5:**
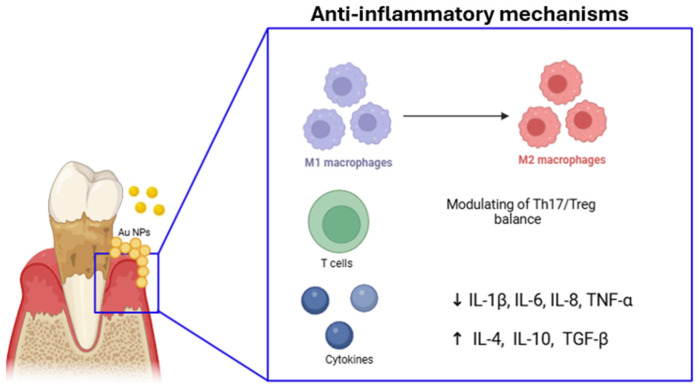
Nanoparticles (NPs) exhibit anti-inflammatory properties in periodontal disease by promoting a phenotypic shift in macrophages from the pro-inflammatory M1 state to the anti-inflammatory M2 state. This transition is characterized by a reduction in pro-inflammatory cytokines (IL-1β, IL-6, IL-8, TNF-α) and an increase in anti-inflammatory cytokines (IL-4, IL-10, TGF-β). Additionally, metal NPs, such as gold nanoparticles (Au NPs), help modulate the balance between Th17 and Treg cells, further enhancing their anti-inflammatory effects (illustration created using BioRender.com).

Inflammation plays a critical role in the progression of periodontitis, leading to the degradation of the supportive tissues surrounding the teeth. Early detection and effective management of inflammation are essential for achieving the best possible patient outcomes. However, current treatment methods often fall short, highlighting the need for innovative approaches that integrate traditional therapies with advanced technologies [2]. Nanoparticle-based drug delivery systems offer a promising solution by providing precise, targeted treatment for inflammatory diseases. These systems have several advantages, including high drug-loading capacity, controllable sustained release, and the ability to cross physiological barriers. Specifically, nanoparticles can modulate the immune response and reduce inflammation by various mechanisms, contributing to the preservation of periodontal tissue (Table 3, Figure 5). They interact with immune cells, such as macrophages and T cells, influencing their behavior and function to mitigate excessive inflammation and promote tissue repair. It was reported that certain nanoparticles with antioxidant properties can neutralize harmful free radicals generated during inflammation, further protecting periodontal tissues from damage [98]. It was also suggested that nanoparticles are applied to restore a vital process in the cells as autophagy [98,99]. Recently, gold nanoparticles (AuNPs) have been applied to rescue the osteogenic potential of PDLSCs by restoring the inflammation-compromised autophagy–lysosome system [99,100].

### 4.1. Immunomodulatory Action

In periodontitis, a complex interplay of immune cells, primarily macrophages, neutrophils, T cells, B cells, dendritic cells, and osteoclasts, contributes to both the initiation and progression of inflammation and tissue destruction. Therapeutic strategies that modulate the immune response, such as reprogramming macrophages from a pro-inflammatory M1 state to a healing M2 state, could help reduce inflammation and promote tissue regeneration, offering potential treatments for periodontitis without relying on antibiotics [101]. Shi J. et al. created a resveratrol-loaded liposomal system (Lipo-RSV) to improve the delivery and effectiveness of resveratrol in treating periodontitis. The liposomal formulation enhances the stability and bioavailability of resveratrol, improving therapeutic outcomes. Lipo-RSV was found to regulate macrophages in the immune microenvironment of periodontitis. This nanosystem was able to shift macrophages from a pro-inflammatory M1 phenotype to an anti-inflammatory M2-like phenotype, promoting healing. This process was mediated through the activation of p-STAT3 and the downregulation of p-STAT1 [102]. Wang Y. et al. introduced quercetin, a known antioxidant and anti-inflammatory compound, onto nano-octahedral ceria, creating a quercetin-loaded ceria nanocomposite (CeO_2_@QU). This nanocomposite synergistically regulates immune responses in periodontal disease by both inhibiting M1 polarization and promoting M2 polarization. The nanocomposite was found to effectively modulate macrophage polarization in in vitro models by increasing the M2/M1 ratio of macrophages after lipopolysaccharide (LPS) stimulation, which mimics bacterial-induced inflammation [103]. Recently, exosome-based drug delivery systems have also been studied [18]. For example, it was observed that the exosome–curcumin complex enhanced the anti-inflammatory effect of curcumin, and due to the natural vehicle (exosome), did not induce immune response, which avoided subsequent side effects [104]. The study found that in patients with periodontitis, there is a destabilized Th17/Treg balance in the peripheral blood, characterized by upregulated Th17 cells and downregulated Treg cells. This imbalance contributes to the chronic inflammation seen in periodontitis [105]. Recently, the exosomes which are nanosized (30–120 nm) were explored for their capacity to alleviate the inflammatory microenvironment by influencing the Th17/Treg balance. Zhang Y. et al. investigated the therapeutic potential of mesenchymal-stem-cell-derived exosomes (MSC-exos) in treating periodontitis, particularly focusing on the benefits of using a 3D culture system to improve exosome production and efficacy (3D-exos). This nanosystem offered a more effective treatment approach for periodontitis by restoring the Th17/Treg balance through the miR-1246/Nfat5 axis [106]. Zheng Y. et al. extracted exosomes from PDLSCs stimulated by *Porphyromonas gingivalis* lipopolysaccharide (LPS). The exosomes from these LPS-stimulated PDLSCs were found to influence CD4+ T cells by modulating the Th17/Treg balance through the miR-155-5p/SIRT1 pathway [107].

### 4.2. Regulating Pro-/Anti-Inflammatory Environment

ROS production is a key biological process in macrophages, particularly when they are activated to kill phagocytosed microorganisms. This process is part of the immune response to infections, where ROS help to destroy pathogens. However, excessive ROS production can be detrimental. When macrophages produce too many ROS, it can push them toward the pro-inflammatory M1 phenotype. This M1 phenotype is associated with the release of inflammatory cytokines and the promotion of chronic inflammation, which can worsen conditions like periodontitis. Thus, while ROS are important for immune defense, their overproduction can contribute to the escalation of inflammatory diseases by driving macrophages to a harmful M1 state [108]. Shi J. et al. observed that a resveratrol-loaded liposomal system (Lipo-RSV) was effective in scavenging reactive oxygen species (ROS) and inhibiting the NF-κB signaling pathway and inflammasomes. As a result, Lipo-RSV reduced the levels of pro-inflammatory cytokines such as IL-1β, IL-6, and TNF-α, which are typically elevated in periodontitis [102]. In animal models of periodontal inflammation, quercetin-loaded ceria nanocomposite (CeO_2_@QU) significantly reduced pro-inflammatory cytokines such as TNF-α and IL-1β and increased anti-inflammatory cytokines such as IL-10, leading to improved therapeutic outcomes [103]. Xuan L. et al. investigated the use of nanoparticle-encapsulated baicalein (Nano-BE) to modulate the pro-inflammatory response in gingival epithelial cells (hGECs). In this study, baicalein (BE), known for its anti-inflammatory properties, was encapsulated in amine-modified mesoporous silica nanoparticles (MSNs), enhancing their solubility and bioavailability. The study showed that this encapsulation improved drug loading efficiency and allowed for a sustained release of the drugs for up to 216 h. Nano-BE treatment significantly downregulated the IL-1β-induced expression of pro-inflammatory cytokines, particularly IL-6 and IL-8, suggesting that Nano-BE effectively modulates the inflammatory response in gingival epithelial cells [109]. Bao X. et al. explored the use of polydopamine nanoparticles (PDA-NPs) as efficient scavengers for ROS in the treatment of oxidative-stress-induced periodontal disease. In this murine periodontitis model, PDA-NPs demonstrated robust antioxidative effects, efficiently scavenging multiple types of ROS, such as superoxide anion (O_2_^−^), hydrogen peroxide (H_2_O_2_), and hydroxyl radicals (OH·) [110]. Non-steroidal anti-inflammatory drug (NSAID)-loaded nanoparticles represent a significant advancement in drug delivery systems, offering a targeted and controlled approach to administering non-steroidal anti-inflammatory drugs. By encapsulating NSAIDs within tiny particles, typically less than 100 nanometers in size, these formulations can enhance drug bioavailability, reduce systemic side effects, and improve therapeutic efficacy. These nanoparticles can be engineered with various materials, including polymers, lipids, and inorganic compounds, each offering unique advantages in terms of drug loading capacity, release kinetics, and biocompatibility [111]. Once administered, these nanoparticles can target specific tissues or cells. They also can be designed to release the drug in a controlled manner, either as a sustained-release or as an on-demand release, optimizing therapeutic efficacy and minimizing adverse effects. They are used for rheumatoid arthritis, osteoarthritis, skin inflammation, and dental pain. In the oral district, NSAID-loaded nanoparticles can be used to reduce pain and swelling after dental procedures [111]. Osorio M.T. et al. developed doxycycline-doped nanoparticles to obtain an anti-inflammatory response on periodontal-ligament-derived stem cells (PDLSCs), which play an irreplaceable role in regeneration of periodontal tissues and maintaining their homeostasis [112]. In this study, NPs were found to be biocompatible and non-toxic for PDLSCs, promoting the differentiation of PDLSCs into osteoblasts and cementoblasts, which are essential for bone and tissue regeneration. They effectively reduced the inflammatory response of PDLSCs, particularly when exposed to inflammatory mediators like IL-1β. Therefore, these doxycycline-loaded NPs could be a promising therapeutic approach for periodontitis thanks to their sustained release of the antibiotic, enhancing tissue regeneration, reducing inflammation, and improving periodontal health [112].

**Table 3:** Anti-inflammatory nanotherapeutic strategies for periodontitis treatment.

Nanoparticles	Study Type	Methods	Effects	References
**Lipo-RSV**	In vitro	Resveratrol-loaded liposomal system	Re-education of macrophages from M1- to M2-like phenotype through activating p-STAT3 and downregulating p-STAT1. Reduction of ROS and inhibition of NF-κB signal and inflammasomes, reducing IL-1β, IL-6, and TNF-α.	[102]
**CeO_2_@QU**	In vitro/in vivo (*P. gingivalis*-infected rat model)	Quercetin onto nano-octahedral ceria	Significant downregulation of pro-inflammatory cytokines and upregulation of anti-inflammatory cytokines	[103]
**3D-exos, 2D-exos**	In vitro	Mesenchymal-stem-cell-derived exosomes produced using 2D and 3D culture systems	Improvement of the function of MSC-exos in the treatment of periodontitis	[106]
**Exosomes**	In vitro	Exosomes from PDLSCs stimulated by *P. gingivalis* and LPS	Influence of CD4+ T cells, by modulating the Th17/Treg balance through the miR-155-5p/SIRT1 pathway	[107]
**Nano-BA, Nano-BE**	In vitro	Baicalin (BA) and baicalein (BE) encapsulated in amine-modified mesoporous silica nanoparticles (MSNs)	Downregulation of IL-1β, IL-6, and IL-8	[109]
**PDA NPs**	In vitro/in vivo (LPS-induced periodontal disease in BALB/c nude mice)	Biodegradable polydopamine nanoparticles (PDA NPs) as smart ROS scavengers	Scavenging of multiple ROS and suppressing ROS-induced inflammation reactions	[110]
**Dox-NPs**	In vitro	Polymeric nanoparticles (NPs) produced by a polymerization/precipitation process and doped with doxycycline	Dox-NPs enhanced PDLSC differentiation into osteoblast/cementoblast lineages while providing an anti-inflammatory effect	[112]

## 5. Nanomaterials for Regenerative Periodontal Therapy

**Figure 6:**
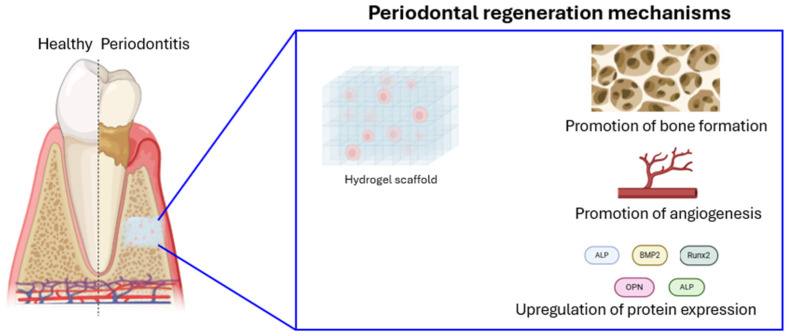
Hydrogel scaffolds create a supportive microenvironment for periodontal regeneration by enhancing bone formation and angiogenesis. This process is associated with the upregulation of key proteins, including BMP2, Runx2, OPN, and ALP, which are essential for tissue repair and regeneration (illustration created using BioRender.com).

Traditional methods for periodontal tissue reconstruction, such as allogeneic and autologous grafts, often present limitations, including limited tissue availability, donor site morbidity, immune rejection, and invasiveness [113]. To address these challenges, nanotechnology offers a promising alternative, as it enables the manipulation of materials at the nanoscale, enhancing their properties and functionalities. Various nanomaterials, such as nanoparticles, nanocapsules, and nanofibers, have shown potential for oral tissue reconstruction and restoration (Table 4, Figure 6) [114]. For instance, calcium phosphate nanoparticles loaded with chlorhexidine, coated with carboxymethylcellulose to enhance bio-adhesion, have demonstrated antimicrobial and mineralizing effects [115,116]. Poly(D,L-lactide-co-glycolide acid) (PLGA)-based nanoparticles incorporating lovastatin and tetracycline facilitate the sequential release of drugs, providing a dual effect of infection control and bone regeneration [117]. Natural polymers like chitosan have further been utilized in core–shell PLGA–chitosan nanospheres encapsulating drugs like simvastatin and doxycycline, promoting periodontium repair in infected areas and enhancing osteogenesis in bone defects [118]. Innovations with materials like Laponite (LAP)-embedded polycaprolactone (PCL) further advance bone regeneration by promoting cellular viability, differentiation, and vascularization [119]. Moreover, nano-hydroxyapatite (nHA) remains a highly researched biomaterial for alveolar bone regeneration due to its promising results in supporting bone tissue repair.

### 5.1. Nanohydroxyapatite (nHA)

Hydroxyapatite (HA) is a widely studied biomaterial in medicine and dentistry due to its excellent biocompatibility and its natural occurrence in hard tissues like bone and teeth. As a significant source of calcium and phosphate, HA is particularly useful for alveolar bone regeneration. However, traditional HA often shows poor mechanical properties due to its porous structure. Nanohydroxyapatite (nHA), on the other hand, displays improved properties. With its smaller particle size, nHA exhibits increased solubility, higher surface energy, and enhanced biocompatibility. This larger surface area also contributes to its excellent bioactivity compared to larger HA crystals [120]. nHA is considered a promising scaffolding material for bone regeneration due to its structural similarity to natural bone. Research suggests that nHA-based scaffolds, such as Gel–nHA, can promote tissue regeneration, making them suitable for endodontic applications [121]. To produce nHA, various synthesis techniques exist, including co-precipitation, wet precipitation, hydrothermal, mechanochemical, hydrolysis, solid-state, and sol-gel methods. Among these, wet chemical precipitation is the most widely used due to its simplicity, reproducibility, and environmentally friendly nature, producing only water as a byproduct [122]. For large-scale and rapid synthesis, microwave hydrothermal methods combined with ultrasonic atomization precipitation offer advantages. This approach produces nHA powders with homogeneous size distribution and excellent dispersibility [123]. In addition, nHA coatings on dental implants, such as stainless steel and titanium, enhance bone integration and new bone formation, leading to improved bone-to-implant contact (BIC) [120]. de Oliveira P.G.F.P. et al. have demonstrated that nHA coatings stimulate cellular activity, including osteoblast and osteoclast activity, and promote bone regeneration [124]. Yamada M. et al. explored the effect of nanopolymorphic crystalline hydroxyapatite (HA) coating on microroughened titanium implants for improving bone–implant integration. The HA coating, created using flame spray and low-temperature calcination, was found to increase surface area and enhance the osteoconductivity of the implants. In a rat model, HA-coated implants showed significant improvements in bone–implant integration, with higher bone contact (50 μm) and volume near the implant surface and reduced soft tissue interference [125]. Additionally, combining nHA with polyacrylamide-based hydrogels (PAAM) enhances post-extraction preservation by supporting osteoblast infiltration, cell adhesion, and fluid retention, while providing strength, degradability, and low cytotoxicity [126]. In oral surgery, nano-polymorphic crystalline HA on titanium surfaces via flame spray and low-temperature calcination boosts bone–implant integration, with improved osteoconductivity localized to the microenvironment [125]. Nano-crystalline hydroxyapatite also binds bone, encouraging osteoblast activity and aiding in bone healing, as evidenced in clinical trials where it supported periodontal tissue regeneration [127]. In studies of nano-HA paste, human periodontal ligament (PDL) cells proliferated in response to the paste, with underlying mechanisms involving the activation of the epidermal growth factor receptor (EGFR) and downstream signaling pathways contributing to periodontal tissue regeneration [128].

### 5.2. Nanostructured Scaffolds for Tissue Engineering

Tissue engineering integrates principles from biology and engineering for restoring, maintaining, or improving tissue function. A common approach involves seeding cells onto a biomaterial scaffold to create functional tissue in vitro, which can then be implanted into a patient [129]. Tissue engineering scaffolds must possess specific properties to facilitate tissue regeneration. They should promote cell adhesion and proliferation, provide a porous structure, degrade at a controlled rate, and provide mechanical support. To mimic the natural extracellular matrix (ECM), scaffolds are often designed with nanofibrous structures, porous architectures, and biomimetic material [129].

### 5.3. Nanofibrous Scaffolds

Nanofiber scaffolds have emerged as a promising technology in the field of tissue engineering, particularly for craniofacial regeneration. These scaffolds, fabricated through techniques like electrospinning, self-assembly, and phase separation, offer a versatile platform for creating biomimetic environments that mimic the extracellular matrix (ECM). By tailoring the fiber diameter, pore size, and mechanical properties, researchers can design scaffolds that support cell adhesion, proliferation, and differentiation [130]. To enhance the bioactivity of nanofiber scaffolds, researchers have incorporated various bioactive molecules, including growth factors, cytokines, and extracellular matrix proteins. These molecules can stimulate cell proliferation, differentiation, and migration, leading to improved tissue regeneration. Additionally, the incorporation of mineral phases, such as hydroxyapatite, can further enhance the osteoconductive properties of the scaffolds [131]. The electrospun nanofibrous membranes of synthetic polymers are widely used in the biomedical field, and polycaprolactone (PCL), polylactic acid (PLA), and poly(lactic acid-co-glycolic acid) (PLGA) are commonly utilized in the treatment of oral diseases [132]. In particular, PCL, a biodegradable and biocompatible polymer, offers excellent permeability, flexibility, and ease of processing. Importantly, it does not generate acidic byproducts during degradation, maintaining the stability of the oral environment [133]. Electrospun PLA nanofibrous membranes are a popular treatment for periodontitis. PLGA nanofibers prepared via electrospinning have gained wide adoption in the treatment of oral diseases, including periodontitis, pulp disease, and tissue regeneration. PLGA-drug-loaded barrier membranes can provide controlled drug release and promote bone tissue growth. Ma et al. observed successful prevention of decreased alveolar ridge height and increased bone growth using PLGA-loaded minocycline (MINO) nanofibers [134]. A novel biodegradable, antibacterial, and osteoconductive electrospun PLGA/PCL membrane was tested as an ideal osteogenic scaffold by Qian Y. et al. [135]. It was structured with serial layers of electrospun chlorhexidine-doped PLGA/PCL, PLGA/PCL, and β-tricalcium phosphate-doped PLGA/PCL. The results suggested that it had superior properties such as higher strength, better cell adhesion, greater osteoconductive properties compared to a single-layer membrane, and antibacterial properties [135]. Recent studies have demonstrated the potential of nanofiber scaffolds for craniofacial regeneration. Xu et al. investigated the use of nanosilicate-functionalized polycaprolactone (PCL/LAP) nanofibrous membranes for periodontal regeneration. The incorporation of Laponite (LAP) enhanced cell proliferation and osteogenic differentiation and modulated the immune responses of periodontal ligament cells (PDLCs), leading to improved bone formation and periodontal attachment in rat models [136]. Kuchler-Bopp et al. explored the use of polycaprolactone (PCL) scaffolds functionalized with poly(lactic-co-glycolic acid) (PLGA) nanoparticles loaded with cyclosporine A (CsA) to promote innervation of bioengineered teeth. The CsA-loaded PLGA nanoparticles, synthesized using a microfluidic method, enhanced innervation within the dental pulp without affecting overall tooth development [137]. Furthermore, studies on nanohydroxyapatite/chitosan/gelatin (nHA/CG) scaffolds seeded with human periodontal ligament stem cells (hPDLSCs) have shown promising results in large jawbone defect regeneration in minipigs. The hPDLSCs adhered well to the nHA/CG scaffolds and significantly enhanced bone formation, suggesting the potential of this approach for future clinical applications [121,138].

**Table 4:** Regenerative nanotherapeutic strategies for periodontitis treatment.

Nanoparticles	Study Type	Methods	Effects	References
nHA	In vivo (diabetic rat model)	Nano-hydroxyapatite (nHA) coating implant surgically placed in tibias	nHA coatings stimulate cellular activity at genetic level of osteoblasts and osteoclast	[124]
nHA	In vivo (male Sprague–Dawley rats)	Nanopolymorphic crystalline hydroxyapatite (HA) coating on microroughened titanium implants	HA-coated implants showed significant improvements in bone–implant integration	[125]
HAp-PADH	In vitro/in vivo (female New Zealand white rabbits)	Hydrogel by a facile one-step PAAm and urethacrylate dextran (Dex-U), followed by the in situ mineralization of HAp nanocrystals	Promotion of osteogenic differentiation of M3CT3 cells, excellent osteoconductivity	[126]
NcHA	In vivo (human)	NcHA bone replacement graft (Sybograf^®^) in combination with bioresorbable collagen membrane (Periocol^®^)	Slight clinical improvement in CAL (clinical attachment level) gain	[127]
nano-HA paste	In vitro	Suspension of pure nanocrystalline HA in water	Stimulation of hPDL proliferation mediated by EGFR and followed by ERK1/2, Akt activation	[128]
MINO-PLGA	In vitro/in vivo (periodontitis rat model)	Minocycline-loaded poly(lactic-co-glycolic acid) electrospun membranes	Sustained diffusion of MINO, good support of osteoblast proliferation and adhesion, increased alveolar crest height	[134]
PPC, PP, PPβ	In vitro	Three-layer membranes structured with serial layers of electrospun chlorhexidine-doped PLGA/PCL (PPC), PLGA/PCL (PP), and β-tricalcium phosphate-doped PLGA/PCL (PPβ).	Better MC3T3 cell adhesion, and promoted osteoconductive properties	[135]
PCL/LAP	In vitro/in vivo (calvarial defect rat model)	Nanosilicate-incorporated PCL nanofibrous membranes	Mediation of osteogenesis and immunomodulation of PDLCs in vitro and accelerating periodontal regeneration in vivo	[136]
CsA-loaded PLGA	In vivo (ICR mouse)	Cyclosporine-A-loaded poly(lactic-co-glycolic acid) (PLGA) nanoparticles	Innervation of 88.4% of the regenerated teeth using the CsA-loaded PLGA scaffold	[137]
Gel–nHA scaffold	In vitro	New gelatin (Gel)–nano-hydroxyapatite (nHA)-based scaffold	Increment of ALP in DPSCs	[121]
nHA/CG	In vitro/in vivo (minipig)	Nanohydroxyapatite/chitosan/gelatin (nHA/CG) three-dimensional porous scaffolds	Adhesion of hPDLSCs, increased new bone formation and generated large bones with normal architectures and vascularization	[138]

## 6. Nanomaterials and Biocompatibility

The biocompatibility of NPs is one of the most critical characteristics that determine whether nanoplatforms are suitable for biomedical applications. For NPs to be effectively used in medical treatments, they must interact safely with biological systems without causing harmful effects, such as toxicity, inflammation, or immune system activation. Their biocompatibility ensures that they can be safely introduced into the body, either for drug delivery, imaging, or tissue engineering, without adverse reactions or long-term harm. This includes factors such as non-toxicity, minimal immune response, biodegradability, and the ability to integrate with the body’s tissues without causing rejection or damage. Consequently, thorough assessment of the biocompatibility of NPs is essential for their successful translation from the laboratory to clinical use in medical and dental fields. There are several surface modification methods available for NPs aimed at optimizing their biocompatibility and enhancing their performance in biomedical applications. These modifications are crucial for ensuring that NPs can interact safely with biological systems, minimizing toxicity and improving their ability to target specific cells or tissues [139]. Polyethylene glycol (PEG), also known as PEGylation, is one of the most widely used coatings for NPs. It creates a hydrophilic, biocompatible layer that helps prevent immune recognition, reduces opsonization (the process by which particles are marked for clearance by the immune system), and increases the stability of the NPs in biological environments [139]. Functionalizing the surface of NPs with biomolecules such as peptides, antibodies, or proteins can enhance their ability to target specific cells or tissues [139]. To reduce the toxicity of NPs, dextran is widely used as a surface modification agent [140]. Dextran is a complex branched polysaccharide derived from glucose, known for its biocompatibility, biodegradability, and low immunogenicity. It is commonly employed to modify the surface of iron oxide nanoparticles (Fe_3_O_4_ NPs), providing several benefits in biomedical applications [70,140]. The findings in the work of Huang Y. et al. suggest the biocompatibility of dextran-coated iron oxide nanozymes (Dex-IONP-GOx) as antimicrobial treatment, supporting its safe application for biomedical purposes. Histopathological analysis of gingival tissues as well as liver and kidney showed no visible signs of harmful effects, such as proliferative changes, vascularization issues, necrosis, or acute inflammatory responses, after treatment [70]. Natural biopolymers like chitosan, alginate, and hyaluronic acid can be also used to coat or modify the surface of NPs. Polymers such as poly(lactic-co-glycolic acid) (PLGA) are often used for this purpose, as they degrade safely into non-toxic byproducts. These polymers are biocompatible, biodegradable, and less likely to induce an immune response. They can also enhance the controlled release of drugs, improve the stability of NPs, and aid in the targeting of specific tissues [139]. Poly(α-hydroxy-esters)-chitosan core–shell nanospheres adopted in different studies not only provide a hydrophilic extracellular matrix–like surface, promoting enhanced cell affinity, but they also enable the sequential release of multiple drugs [117,118]. This unique combination of properties makes them highly suitable for applications in drug delivery systems, where controlled and targeted release of therapeutic agents is essential. The hydrophilic surface mimics the natural extracellular matrix, facilitating better interaction with cells, while the core–shell structure allows for the encapsulation and sustained release of different drugs in a controlled manner, improving treatment efficacy and minimizing side effects. Hydrogels can be incorporated onto the surface of nanoparticles to enhance their biocompatibility by providing a cushioning layer that mimics natural tissue environments. This is especially useful for applications in regenerative medicine and tissue engineering, where the nanoparticles must integrate with biological tissues without causing an inflammatory response. Fang J. et al. developed a strong, tough, osteoconductive hydrogel through a simple one-step micellar copolymerization of acrylamide and urethacrylate dextran (Dex-U), followed by the in situ mineralization of hydroxyapatite (HAp) nanocrystals. The biocompatibility of the HAp-PADH hydrogel is attributed to its hydrophilic surface, the use of biocompatible materials like dextran and hydroxyapatite, and its ability to support cellular functions such as osteoblast proliferation and differentiation [126]. These properties not only reduce the risk of toxicity or immune rejection but also enhance its potential for use in bone tissue engineering and other regenerative medical applications. Overall, while surface modifications are widely used to enhance the biocompatibility, stability, and functionality of NPs, the exact mechanisms by which these coatings interact with biological systems are still not fully understood. Improving the specific interactions of various surface coatings, as well as refining their delivery mechanisms, continues to be an ongoing area of research. Advances in this field are crucial for optimizing the targeted delivery, controlled release, and overall safety of nanomaterials in biomedical applications.

## 7. Challenges and Future Perspectives

One of the primary challenges with nanomaterials is ensuring biocompatibility. Some nanoparticles may trigger immune responses or cytotoxicity, leading to inflammation, irritation, or damage to healthy tissue [141]. More importantly, the behavior and properties of metals change significantly from the micro to the nano size, and therefore their potential toxicity at the nanoscale must be deeply investigated [142]. Addressing these issues requires comprehensive surface modifications and functionalization with biocompatible molecules to enhance their safety profile. The long-term effects of nanomaterials in the oral cavity are still not fully understood, making it important to evaluate their safety before widespread use. Nanomaterials used in periodontal treatments must be stable in the oral environment, where factors like saliva, enzymes, and the presence of biofilms can degrade materials or alter their properties. To address these challenges, researchers are developing nanomaterials with specific properties to enhance stability in the oral environment. For example, hydrophilic coatings can help resist enzymatic degradation, while hydrophobic coatings might resist bacterial attachment [117,118,139]. Furthermore, the delivery of nanomaterials across the biofilm or infected plaque, where bacteria reside in a protective matrix, can be difficult. Nanomaterials must be able to penetrate these biofilms and interact directly with the bacteria-deriving biofilm, leaving unaltered health tissues. Inorganic nanoparticles (NPs) with enzyme-like properties called nanozymes are designed to mimic the activity of natural enzymes without relying on complex biological machinery. Nanozymes are promising in overcoming the above limitations, by degrading the key components of oral biofilm, which weakens the biofilm structure and facilitates the penetration of therapeutic agents [69]. They catalyze reactions through mechanisms like oxidation, reduction, hydrolysis, and metal ion coordination, based on their size, shape, surface charge, and functionalization [69]. For instance, Zhang et al. developed DNA nanozymes for detecting dental bacteria, while Huang et al. created iron oxide nanozymes to selectively inhibit biofilm deriving from *Streptococcus mutans* [143,144]. Achieving precise targeting of nanomaterials to the infected tissues is essential for maximizing the effectiveness of treatment. While nanocarriers can deliver drugs or bioactive molecules directly to the site of infection, challenges remain in controlling the release profiles and preventing premature degradation or drug leakage. For instance, some nanoparticles may lose their functional properties over time, reducing their effectiveness in sustained drug release or antibacterial action. Ensuring the long-lasting effectiveness of nanomaterials is essential for providing therapeutic benefits over the course of treatment [145]. The use of nanomaterials in medical applications is still a relatively new field, and regulatory frameworks for their approval in periodontitis treatments are still developing. Regulatory bodies, such as the FDA or EMA, require rigorous testing to ensure that these materials are both safe and effective for human use [142]. There are also ethical concerns surrounding the potential unintended consequences of using nanomaterials, especially regarding long-term health impacts and the environmental consequences of widespread nanomaterial use. Nanomaterials can persist in soil, water, and air, potentially influencing ecosystems; however, the green chemistry approach focusing on environmental concerns, efficiency, and economy seeks to reduce this risk [142].

Future research should focus on long-term in vivo studies in animal models to evaluate the safety and efficacy of nanoparticle-based scaffolds, as well as exploring synergistic effects with other bioactive molecules or growth factors to further enhance bone regeneration therapies. For example, the use of stem cells in combination with nanomaterials raises several regulatory and ethical concerns. The safety and efficacy of stem-cell–nanomaterial combinations must be rigorously evaluated. Regulatory bodies, such as the FDA and EMA, require comprehensive preclinical and clinical testing to ensure that these treatments do not pose undue risks, such as immune reactions, tumor formation, or unintended biological interactions. The future of nanomaterial-based therapies for periodontitis lies in improving the targeted delivery of antibacterial agents, anti-inflammatory drugs, or growth factors directly to the sites of infection. Nanoparticles can be functionalized to selectively bind to specific bacterial strains or tissue receptors involved in inflammation. The development of smart nanomaterials capable of responding to changes in the local environment (such as pH, temperature, or enzyme activity) could offer on-demand drug release, ensuring that therapeutic agents are only released when needed and at the right concentration. Nanomaterials with inherent antibacterial properties, such as silver nanoparticles, copper oxide nanoparticles, or graphene-based materials, are being explored for their ability to kill bacteria directly. These materials can be incorporated into toothpastes, gels, or mouthwashes to provide localized, sustained antimicrobial activity. Advances in nanotechnology could lead to personalized periodontal treatments based on an individual’s specific bacterial profile. For example, nanomaterials could be tailored to target particular pathogens or inflammatory pathways specific to a patient’s condition, improving treatment efficacy and reducing side effects. Nanomaterial-based sensors embedded in dental implants or as part of wearable devices could enable real-time monitoring of oral health and bacterial activity. These sensors could be used to track early signs of infection, allowing for proactive interventions before periodontitis progresses. While significant progress has been made in the development of nanofiber scaffolds for craniofacial regeneration, several challenges remain. These include the need for improved control over fiber alignment and pore structure, the development of scalable manufacturing processes, and the long-term evaluation of biocompatibility and efficacy.

## 8. Conclusions

Nanomedicine holds transformative potential for the future of dentistry and periodontal therapy, offering more effective, targeted, and minimally invasive treatment options. With the continued development of nanotechnology, it is expected to revolutionize the way dental professionals prevent, diagnose, and treat a range of oral conditions, especially periodontal diseases. However, their successful application in clinical settings will require addressing key challenges related to biocompatibility, targeted delivery, stability, and cost-effectiveness. While nanoparticles hold immense potential in revolutionizing dentistry through their diagnostic, therapeutic, and regenerative applications, addressing key challenges is essential for their broader adoption. Cost-effectiveness remains a critical concern, as the synthesis and functionalization of nanoparticles can be expensive, limiting their accessibility. However, nanozymes represent a good example of cost-effective nanomaterials. Indeed, nanozymes are cost-effective, stable, easy to synthesize, and efficient compared to natural enzymes. Additionally, achieving regulatory approval involves rigorous safety and efficacy evaluations, which can be time-consuming and complex. Overcoming these challenges will require collaborative efforts in research, innovation, and policymaking to ensure that nanoparticle-based solutions are not only effective but also affordable and widely available for clinical use. The future of nanomaterials in periodontitis treatment lies in the development of smart, responsive systems that provide personalized, localized, and non-invasive treatment options for patients, ultimately contributing to more effective and efficient management of periodontal disease. While research and clinical trials in humans are still in the early stages, there is growing evidence that nanomedicine can offer significant benefits for the treatment of periodontal diseases. To further advance the practical application of nanotechnology-based systems, it is essential to explore several key avenues of research. First, long-term clinical trials are needed to assess the safety, efficacy, and potential toxicity of these systems in real-world settings. While preclinical studies often show promising results, the translation of nanotechnology into clinical practice requires robust data from extended trials to evaluate long-term effects and to ensure consistent performance across different patient populations. Second, real-world testing of nanoparticle-based systems is essential to determine their behavior in complex biological environments, as laboratory conditions often differ from the dynamic conditions in human tissues. This includes understanding how surface coatings affect nanoparticle distribution, immune response, and clearance over time, as well as how they interact with other drugs or medical devices in clinical use. Lastly, personalized nanomedicine will benefit from deeper exploration into the customization of surface coatings for specific patient needs, considering factors such as genetic variability, disease stage, and tissue-specific targeting. Interdisciplinary collaboration among material scientists, biologists, and clinicians will be key to optimizing these systems for patient-specific therapies. By focusing on these future research directions—long-term clinical trials, real-world testing, and personalized approaches—the potential of nanotechnology-based systems can be more effectively realized, paving the way for their successful integration into mainstream medical treatments.

## Data Availability

Data are contained within the article.
